# Clinical Cases

**Published:** 1882-02

**Authors:** E. W. Berridge

**Affiliations:** London


### CLINICAL CASES.

E. W. BERRIDGE, M.D., LONDON.

*Phosphorus in Rheumatism, etc.* Mrs. A. æt, 35, Oct. 2nd, 1871. Left arm painful for six weeks. Numbness and feeling of "pins and needles" in left forefinger, extending up radial side of hand and wrist. First phalanx of left thumb feels, at times, as if in a vice; gnawing pain in left fore-finger, extending when severe up radial side of arm to elbow, where the bone feels as if it would come through; sensation of heat in these parts, worse at night. The gnawing seems to be in the bone. The gnawing and burning are relieved by lying on back or leaning back; on letting the left arm hang down, she feels a rush of blood to the left fore-finger, and pain as if the nail were being pulled out. Menses scanty, regular; during menses and for fourteen days before menses, much flatulence, rolling in abdomen, causing great pain, relieved by bending double and by hot drinks; the last menses were last week. These menstrual symptoms she has had for eight or nine years. After drink, ing, especially warm drink, the face and arms become red; warm drinks make her feel as if she could not get her breath. These dyspeptic symptoms she has had for eighteen months. The first and second left finger swell for three hours every day. First left finger is contracted, cannot straighten it except by the aid of the other hand; pain in the hand prevents sleep; bruised feeling at root of left thumb. Dislocated pain in left knuckles and wrist, extending as far as the elbow, for the last three weeks. Is obliged to carry left arm in a sling. For the last month has been under allopathic treatment, taking *Nitre, Belladonna, Opium* and *Chloral*, but without relief.

*Diagnosis of the Remedy.*—The latest symptoms, and therefore. *cœteris paribus*, the most characteristic of the remedy, were the pains in the left upper extremity. Bœnninghausen's Repertory gave the following:—

Upper extremity worse by letting arm hang down: *Angust. Cina, Ignat., Magnes. aust., Nat. mur., Nux, Paris, Phosph., Phos. ac., Plat., Puls., Ruta, Sabina, Stront., Sulph. ac., Valer.* Gnawing pain in upper extremity (various parts): *Alum, Cantharis, Dros., Dulc., Graph., Laur., Mag. carb., Mangan., Phosph., Ranun. scel., Stront., Sulph.*

This reduces the list to *Phosph.* and *Stront.* Of these *Phosph.* alone has the constriction of fingers, the rush of blood to the hand, the swelling of fingers, the bruised pain in hand, and the dislocated pain in fingers (all these given in Bœnninghausen's Repertory). *Phosph.* has also aggravation from warm food (Bœnninghausen's Pocket-Book). The menstrual symptoms were not apparently covered by the remedy, but as these were of old standing, they were not of so much diagnostic value. Accordingly, I gave her one dose of *Phosph.* CM (Fincke).

Oct. 9th. Numbness, rush of blood to the finger, and bruised pain unchanged; pain in first phalanx of thumb, pain in wrist and knuckles, and pain in nail better; less dyspnœa after warm drinks; slept better; has stiffness of forearms; no burning, but the place which used to be hot feels sore to touch; arms no longer red after drinking, face very slightly so; no return of swelling till three days ago; the gnawing extends up to left shoulder and left neck; it is relieved by placing the arms over head at night.

Oct. 16th. Numbness unchanged; pain in first phalanx of thumb is gone; the gnawing is only in shoulder and elbow: the burning has returned for six days; the rush of blood less for five days; less flatulence before menses, lasting only two days and with less pain; the symptoms from warm drinks returned last night; bruised pain and dislocated pain better; pain in nail better till yesterday, when it returned; the swelling has occurred only twice; the pain in elbow is like grating the bone; has been generally better till yesterday, has not needed the sling for a week; gnawing still in left neck; yesterday, sneezing and running from nose, worse out of doors, with burning in the eyes (effect of *Phosph.?*).

Oct. 23rd. Numbness, rush of blood and pain in nail gone; gnawing from middle of arm to shoulder, the swelling and the dislocated pain in wrist worse; scraping in elbow bone; forearm and hand feel burnt, as if skin were off; the aggravation from warm drinks only at times, it used to be constant; bruised pain still; straightens fingers more; pain in neck on bending head backward.

Oct. 31st. Pain only in elbow bone, which feels sore and stiff; burnt feeling nearly gone; no dislocation pain for five days; no gnawing for a week; can play the piano; menses returned last week; the aggravation from warm drinks returned for five days; all other pains gone; can straighten finger; felt tired for five days and sleepy in morning, and after sleep does not feel refreshed.

1872, May 14th. Reports that about a month after last visit the dyspnœa on drinking ceased; in two months the redness of face and arms and flatulence ceased. The other symptoms ceased in eight days.

*Comments.*—The latest symptom—the pains in the upper extremity were the first to be removed, as Hahnemann teaches; and though some of the older symptoms were ameliorated at the same time as the later ones, they returned before they finally ceased. The dyspnœa, which was the next symptom in point of duration, was the next to be removed; and the menstrual flatulence, which was the oldest symptom, was the last to disappear. It is true that the redness after warm drinks, which had not existed so long as the flatulence, disappeared at the same time: but the fact that they were both connected with the digestive system, and so inter-dependent on each other, may account for this apparent irregularity in the order of their departure.

*Belladonna in Nervous Affection of the Throat.*—Mr. H., an artist, consulted me, Sept. 13th, 1871. Two years previously had irregular meals and smoked much; at that time he had a sensation in throat as if throttled, which recurred on mental worry, as if he could not swallow; fear of being in company in a room; these symptoms lasted four or five months; then *after perfect rest*, and taking allopathically, bark and valerian, they ceased for five or six months; they then returned, and have now lasted eleven months.

*Present symptoms.*—Feeling as if he could not swallow, caused by any mental worry, which produces also a feeling as if he could not remain in company; this comes on when fasting, but is removed by food; when he leaves off smoking, he gets irritable with every one; he has been gradually leaving off smoking. At night lies awake thinking of the business of the day. When the throat sensation comes on he feels very angry with himself, and loosens the clothes from the throat; cannot bear noise; the nervous symptoms are always relieved by alcohol; drinking water relieves the throat sensations; he must completely swallow one drink before he takes a second, otherwise it goes up to the posterior nares, and the back of the throat then feels constricted; when the sensation comes on he must stand still; fear of going out of doors; cannot swallow solids without drinking; six months ago difficulty in swallowing liquids, the same again lately; swallowing saliva is difficult; constant desire to swallow, with feeling as if he would suffocate if he did not; the choking is excited by the repetition of scenes which had formerly caused it; pain at occiput as if a string were tied there; throat feels dry; the dysphagia is worse when he thinks about it, better if he thinks of something else; throat feels contracted. For a year has had warts on hands, first on right then on both. Before the throat symptoms first came on, he had itching red pimples in axilla and upper arm, removed by applications of vinegar and other lotions.

*Diagnosis of the Remedy.*—The most characteristic symptom, because occurring under the fewest remedies, was the sensitiveness of the throat; taking this as the starting point, I found the following in Bœnninghausen's Repertory and Pocket-book:—sensitiveness of the throat: *Bell.*, *Laches.*, (*Phosph.*) *Sulph.*: Relief from alcohol: *Bell.*, *Lach.*, *Phosph.* (and others not possessing the preceding symptom).

Warts: *Bell.*, has the first rank; *Lach.*, and *Phosph.*, being in the fourth. *Bell.* corresponded also fairly with the other symptoms, so I gave him one dose of *Bell.* 60 M (Fincke), and advised him to gradually discontinue smoking.

Sept. 20th. Has been much better; more able to think; only one throat attack, which was on 17th; no more fear of going out; enjoyed a long walk last night, more than he has done for years; the pain in the head has occurred once or twice, but is considerably less; slept very well last week; can bear noise better: feels decidedly better generally.

Sept. 27th. Feels better; has had several throat attacks, but less often than before; can walk better; sleep good; has had no throat attacks indoors; when he first consulted me, he had them at all times; they are less strong when they do occur; less depressed in spirits; was over-fatigued last week.

Oct. 4th. Much better; only three or four attacks; has been hard at work and can do it well; the last attack was on the 2d; they are shorter and less severe than formerly; can walk well; very much less depressed; painting no longer brings on the attacks as it did when I first saw him.

Oct. 16th. Only three or four attacks, shorter and slighter, more when indoors; has felt "wonderfully well" since last visit; has none of the nervous feeling which used to prevent him from going out of doors; he still smokes, but less.

Oct. 24th. No more attacks, though inclined thereto at times; has been to a theatre without bad effect; has smoked rather more; says he feels quite different since he has consulted me.

Nov. 10th. Has been a long railroad journey, had no attacks in the train, but afterwards had two or three from worry; since last visit has had only three or four attacks altogether; has smoked more; smoking causes more dryness of mouth and bodily depression than it did.

Nov. 27th. Has remained quite well till yesterday, when he had an attack for ten minutes from worry; has not been free for so long a time before.

Dec. 13th. No more attacks, but once or twice a threatening; has not diminished smoking.

1872, Feb. 13th. No more attacks, but a threatening of them at times; has been much worried for some weeks.

April 13th. Has been quite well till two weeks ago; has had much worry and mental work; has had once or twice a slight return of throat symptoms; on swallowing food, it feels as if it would not go down; appetite not good; has not left off smoking, as the action of the dose (which had now lasted *for two hundred days*) seemed exhausted, and as there were no new symptoms, which generally indicate another remedy, I gave him another dose of *Bell.* 60 M (Fincke).

May 18th. No more attacks; appetite better; warts on hand going away for a week. (He drank claret last week; could this have affected the warts, or was the action of the first dose expended on the internal symptoms?)

June 30th. Has had but little throat irritability; must swallow his food slowly when he thinks of it; warts almost gone.

1873, May 20th. No return of symptoms to this day. The action of the single dose of the high potency was not checked by smoking, even though it produced symptoms of *Tobacco.*

*Cocculus in abscess, etc.*—1873, Aug. 14th, Mr. H., a youth about 14 years old, consulted me at 9.30 P. M. Since 2 P. M. has had a superficial abscess just above root of nail of right little toe, with pain as if a hot iron went into it on raising the foot from the ground in the act of walking; it causes very great difficulty in walking. For a week, three or four watery stools a day, though there would be a dozen if he yielded to it; none in night, but it comes on directly after rising from bed, so that he has scarcely time to dress; standing increases the diarrhœa, sitting stops it; with the stool, pain in bowels which causes dyspnœa, sweat, and faintness. Objects seem to move up and down. To-day, when reading writing, could only see the left half of the lines.

*Diagnosis of the remedy.*—The latest symptom was the abscess, therefore, the most important in the selection of the remedy. Under abscess of the toes, Bœnninghausen gives *Cocculus* only, and though it did not correspond to the other symptoms, it was the most suitable remedy, at least to commence with, on account of the quality of the symptom indicating it; this symptom being not only the most recent, but also the most peculiar, belonging to only one remedy in the whole materia medica. I gave him one dose of *Cocculus Indicus* CM (Fincke), and instructed him to repeat the dose after every diarrhœic stool. Perfect rest, and a poultice (unnecessary) to the toe.

Aug. 15th. Felt slight urging this morning, and took a second dose. In evening toe was nearly well, abscess dried up without discharging. No more diarrhœa, but natural stool. Sight natural. Very little pain or faintness. Soon recovered.

This potency of *Cocculus* was prepared by Dr. Fincke, from a tincture which I sent him, made in India from the fresh berries. The case adds several new clinical symptoms to the materia medica of this remedy; it also shows that pus can be absorbed under the action of the homœopathic remedy, without evacuation.

*Borax in vertigo.*—For 12 years, Mr. W. has had at times attacks of vertigo, *as if pushed from right to left* and somewhat forwards; the attacks have been troublesome for a month or so. *Borax* is the only remedy wnich has produced the italicized symptom; so I gave him one dose of C M (Fincke). The attacks ceased at once, and he had only two or three afterwards, arising from great mental exertion or worry. Cured

*Antimonium crudum in urticaria.*—1871, Aug. 9th, a boy had been subjected to urticaria all over his body for 15 months, *white lumps with red areolæ* and itching. The longest interval of freedom has been three weeks, often only one. It is worse after meat; makes him irritable, very hot and thirsty. His mother had urticaria when pregnant with him. I do not think I ever saw *Ant. crud.* fail in urticaria when characterized by the italicized symptom. I gave him one dose of 200 (Lehmann).

Oct. 11th. Reports only slight rash last week.

1872, April. No return of the symptoms.

*Turpentine in paralysis.*—April 27th, 1881, Mr. P., æt. 38. Has suffered for 4 years from general paralysis. He has been during this time under the care of a most skillful Hahnemannian, who from time to time greatly relieved him. But as his condition now required that he should be visited, my friend asked me to see him, as he lived in my neighborhood. He had been getting weaker and confined to his bed for three weeks. I found him with some horrible bed-sores, one on each tuber ischii, one on lower part of sacrum, and one on right hip; they were black, bleeding, discharging offensively, and painful on moving. According to the indication given, page 266 of C. Lippe's invaluable Repertory, I gave *Sulph. acid* CM (Fincke), a dose three times a day in water.

May 5th. In three days the bed-sores became drier, and less offensive. He has slept much better. He now tells me of another symptom which he has had for 14 months,—intense pains in the legs. He was unable to define these pains so as to afford a basis for treatment, so I resorted to the earlier symptoms of the case. I ascertained that the paralysis commenced in the right arm and left leg *at the same time;* a year afterwards it went to the right leg. To my interleaved copy of Lippe's Repertory I had added, page 303, "Paralysis of right arm and left leg. *Terebinth.*" The source of this symptom I cannot now call to mind, but it is probably from a case of poisoning in some allopathic journal. I gave the patient

*Terebinthina* 200, three times a day. It speedily and permanently removed the severe pains, and in 11 days he died with complete euthanasia. This case illustrates the importance, in some instances, of the early symptoms of the disease. Diagonal symptoms, such as "upper right and lower left," are always of great value.

*Pulsatilla in the pains of real and spurious pregnancy.*—Mrs. P., in the ninth month of pregnancy, complained of troublesome pains of a vague character. She had suffered from them in a former pregnancy, but her mongrel physician failed to relieve her. There was absolutely nothing characteristic in the symptoms themselves. I therefore selected the anatomical region as the key-note, and as *Puls.* has an especial action on the uterus, particularly the pregnant uterus, I gave her a very high potency thereof. The pains were promptly removed by it; and comparison of the two modes of treatment were made by the patient, by no means to the credit of her former medical attendant. In case of false pregnancy, *Puls.* in a very high potency promptly removed some vague but troublesome pains (compare case of false pregnancy cured with *Puls.* 30, reported in *The Organon*, vol. 3, p. 270).

These cases illustrate the fact that the *anatomical region chiefly or primarily affected* may at times be the keynote in the selection of the remedy. The symptoms of a patient may be mathematically compared to a cube. A cube has six sides. The six sides of the diseased condition are, (1) the *cause* (e. g. *Acon.*, for symptoms from cold dry air); (2) the *character* of the symptoms (e. g. *Apis* in stinging-burning pains); (3) the *conditions* (e. g, *Lycop.*, in aggravation from 4 to 7 P. M.); (4) the *concomitants* (e. g. *Castoreum* for yawning in connection with abdominal troubles); (5) the *chronological order* of the symptoms (e. g. the latest symptoms are, *cœteris paribus*, of the most importance; also the mental symptoms which are the last to be developed, the organism repelling the disease from the mind as long as possible); and (6) the *anatomical region* (illustrated in these two cases).

Each of these sides of the cube needs to be considered in every case, and either of them may be the keynote or starting-point in the selection of the remedy. But to base a system of therapeutics on one of these sides only, is as absurd as to imagine that one side of the mathematical figure constitutes the cube. Yet this has been done in the so-called system of Organopathy, a rehash which has not even the poor merit of originality, and which is only exceeded in ignorant foolishness and impudent audacity by the still more recent system of Kidd–opathy.

